# Doctors are striking for a pay-rise, but better conditions are needed to make them feel valued. Key insights from New Zealand

**DOI:** 10.1016/j.fhj.2024.100012

**Published:** 2024-02-19

**Authors:** Michael Clark

**Affiliations:** Internal Medicine Trainee, Rheumatology Department, Queen Elizabeth University Teaching Hospital, Glasgow, UK

**Keywords:** Doctors strike, New zealand, Working conditions, Meals, Study leave

## Abstract

Degradation of junior doctors pay has led to strikes in the UK. Improved pay is important, however to feel valued at work, doctors must have good conditions as well. The author describes how their experience working in New Zealand (NZ) highlighted several insights into how better working conditions make doctors feel more valued.

Three factors are discussed which improve doctors daily experience. Firstly, the use of ‘relief doctors’, who cover inevitable absences caused by sickness or holiday, allow maintenance of proper staffing levels. Secondly, NZ doctors get 6 weeks of paid study leave to prepare for exams, whereas in UK this does not exist. Lastly, in NZ meals are provided whilst at work, this is not the case in the UK.

NZ demonstrates appreciation for its doctors through real and tangible improvements to their working environment. In the UK, pay needs to be improved, but so do working conditions.

## Background

Junior doctors in England have been forced to strike over a real-terms pay cut of 23.5% over the past 15 years.^1^ In Scotland, industrial action has been avoided through an improved pay offer. The degradation of doctors pay shows that the government values doctors less in 2023 than they did in 2008. A doctor is literally worth less now. Pay is important, however to feel valued at work doctors must have good conditions as well. I have recently returned from working as a junior doctor in New Zealand (NZ) for 2 years. My experience there has given me insight into how better working conditions make doctors feel more valued.

Annually around 450 doctors from the UK registered with the NZ medical council, and of these about one fifth will stay long term.^2^ Around 3,000 UK graduates now make up a sixth of the 18,000 doctors working in NZ.^3^ Compared to the UK, NZ doctors work similar hours for similar pay, so in my view pay does not fully explain why UK doctors want to leave. One contributing factor for this migration, could be poor conditions and morale in the NHS. The 2022 General Medical Council National Survey shows that half of UK junior doctors are feeling emotionally exhausted at work.^4^ So how does NZ create a better working environment for its doctors?

## Systemic resilience

Learning to deal with stress and challenge has become a key attribute for any doctor working in the NHS. ‘Resilience’ has become the buzzword to encapsulate this idea. I've had numerous lectures and training sessions on developing resilience. From reflective writing to playing sports and learning instruments, many different methods were suggested for coping with stress. This understanding of resilience has always seemed flawed to me. The onus of resilience should be on the system rather than on the individual. A resilient system should have built in redundancy.

As an example, in NZ, at any time there is a team of doctors working in ‘relief positions’. Their role is to cover absence caused by sickness, leave or nightshifts. This resilient system allows for teams and wards to remain fully staffed during inevitable periods of doctor absence. By allowing teams to remain fully staffed, pressure is reduced on the remaining members and best patient care can be maintained. The knowledge that relief doctors will cover time of absence allows NZ medics to take sick leave when unwell without guilt that the ward may left short staffed, as is often the case in the NHS. In the UK locum doctors are employed to fill gaps, these however add extra expense to an already financially stretched system, so are usually saved for the cover of essential services out-of-hours.

## Study leave and exams

In both UK and NZ there is funding and leave made available for doctors to attend conferences and courses. However, there is an important point of difference when it comes to exams. At several stages in a medical career, exams are required for career progression. These periods are immensely stressful given the amount of studying required to pass. In the NHS paid study leave for exam preparation does not exist. Most NHS doctors therefore have no choice but to use annual leave to prepare for exams, also studying in their ‘spare’ time out of working hours. This is particularly challenging for those with families and dependents. By contrast, in NZ those in training programmes are entitled to 6 weeks study leave prior to exams.^5^ Given that there is a roster of relief doctors in NZ their absence from the ward is covered. This means in NZ doctors feel supported when sitting their exams. The system of support built into the NZ system allows for workforce development, education and therefore progression. This resilient workforce may experience less burnout and professional disengagement, allowing for progression to senior roles earlier, and benefitting the system as a whole.

## Meals

Junior doctors in NZ are entitled to free meals when at work.^5^ The benefits of this simple sounding arrangement goes beyond providing tired doctors with a square meal, it gives junior doctors a sense they are valued and cared for. The fact that the NHS no longer supplies meals at work sends the opposite message to staff. In fact hospitals have had to campaign for access to hot food at night,^6^ although many remain with cold vending machines sandwiches.

## Conclusion

New Zealand demonstrates appreciation for its doctors through real and tangible improvement to their working environment. In the UK there is rhetoric about valuing doctors, but unfortunately this does not translate to the changes needed now.

## Declaration of competing interest

No Conflict of Interest from author.
